# Stress, strain, and pregnancy outcome in postpartum cows

**DOI:** 10.21451/1984-3143-AR2019-0063

**Published:** 2019-10-23

**Authors:** Matthew C. Lucy

**Affiliations:** Division of Animal Sciences, University of Missouri, Animal Science Research Center, Columbia, MO, USA.

**Keywords:** stress, strain, pregnancy, cattle

## Abstract

Stress affects the productivity and fertility of cattle. Stress causes strain and individual animals experience different amounts of strain in response to the same amount of stress. The amount of strain determines the impact of stress on fertility. Typical stresses experienced by cattle include environmental, disease, production, nutritional, and psychological. The effect of stress on the reproductive system is mediated by body temperature (heat stress), energy metabolites and metabolic hormones (production and nutritional stresses), the functionality of the hypothalamus-pituitary-gonadal (HPG) axis and (or) the activation of the hypothalamus-pituitary-adrenal (HPA) axis. The strain that occurs in response to stress affects uterine health, oocyte quality, ovarian function, and the developmental capacity of the conceptus. Cows that have less strain in response to a given stress will be more fertile. The goal for future management and genetic selection in farm animals is to reduce production stress, manage the remaining strain, and genetically select cattle with minimal strain in response to stress.

## Introduction

**Figure 1:**
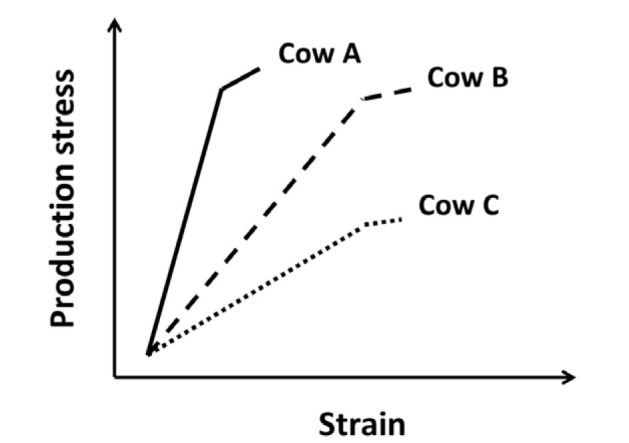
Graph depicting the relationship between production stress and associated strain. For cow A, an increase in production stress leads to the smallest increase in strain. Cows B and C have progressively greater production strain in response to production stress. The most desirable cow is A because there is the least strain in response to production stress.

The correct definition of stress is important in any discussion of reproduction and pregnancy in cattle. A *stress* or *stressor* is a force external to a system that acts to displace the system. A stress condition can be quantified and applied equally across animals. A *strain* is the animal’s response to stress (the magnitude of the displacement). The strain often represents a cost to the individual animal. As depicted in Figure 1, the level of strain in response to an equivalent stress can vary from animal to animal. There is production stress, for example, that places strain on the animal (Fig. 1). The strain in response to greater stress is minimally increased in cow A, moderately increased in cow B, and greatly increased in cow C (Fig. 1). The goal for future management and genetic selection in farm animals is to reduce production stress, manage the remaining strain and genetically select cattle with minimal strain in response to production stress (Cow A).

**Figure 2:**
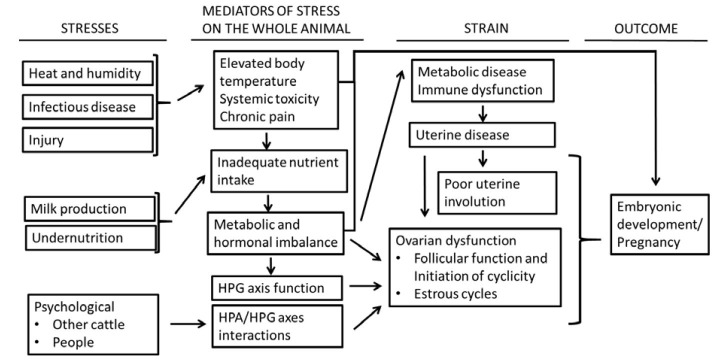
Diagram depicting stresses that affect cattle, the mediators of stress in the whole animal, and the associated strain that ultimately affects pregnancy outcome. HPG = hypothalamic-pituitary-gonadal; HPA = hypothalamic-pituitary-adrenal.

Stressors assume a variety of forms (Fig. 2). The stresses create strain that can affect many aspects of animal production including embryonic development and pregnancy outcome. Cows that have less strain in response to a stress will be more fertile (cow A). Less strain may be explained by lesser biological response to stress or a greater capacity to function (cope) in the presence of stress. Stresses, their mediators, and strains that affect pregnancy in cattle are reviewed in this paper. The stresses are described first, followed by the mediators of stress, the strains, and finally the outcome (effect on embryonic development and pregnancy outcome).

## Stresses that affect pregnancy outcome in cattle

There are many well-known stresses that affect the productive and pregnancy outcome of cattle. The reader is referred to several recent review articles on stressors and the mechanisms through which stress can affect production and pregnancy outcome (Tab. 1). This review will attempt to integrate some of the difference stressors and their effects on pregnancy.

**Table 1:** Table 1. Sources of stress and recent review papers written on the topic.

Sources of stress	Review paper
Infectious disease of the reproductive tract	Bromfield *et al.*, 2015; Eckel and Ametaj, 2016; Sheldon *et al.*, 2018; Gilbert, 2019.
Injury	Funnell and Hilton, 2016; Jewell *et al.*, 2019; Kammel *et al.*, 2019.
Heat	Gwazdauskas, 1985; Hansen, 2007, 2009; Collier *et al.*, 2017; Polsky and von Keyserlingk, 2017; Roth, 2017, 2018a.
Metabolic imbalance postpartum	Lucy *et al.*, 2014; Baumgard *et al.*, 2017; Overton *et al.*, 2017; Lucy, 2019.
Social/psychological	Dobson and Smith, 2000; Karsch *et al.*, 2002; Breen and Karsch, 2006; Ralph *et al.*, 2016; von Keyserlingk and Weary, 2017.
Nutritional	Butler, 1998; Wilde, 2006; D’Occhio *et al.*, 2019.
Transportation	Hong *et al.*, 2019.

### Heat and humidity

Environmental stress caused by heat and humidity (heat stress) is common in both beef and dairy cattle but the strain (elevated body temperature) associated with heat stress is greater in dairy cows (Collier *et al.*, 2017; Polsky and von Keyserlingk, 2017). Lactating dairy cows are particularly sensitive to heat stress because there is metabolic heat production associated with high milk production. In a study of Florida dairy cows, for example, summer infertility was greatest in high milk producing dairy cows (al-Katanani *et al.*, 1999). There is an additive negative effect, therefore, of heat stress and greater milk production on pregnancy outcome in dairy cows. The effects of environmental stress on lactating cows is explained partially by reduced feed intake but there are aspects of the metabolic response that are entirely unique to heat stress (Baumgard and Rhoads, 2013). Many breeds of European cattle are not adapted to production in hot and humid environments. Crossing European breeds with local indigenous breeds will yield lactating cattle that are resistant to heat stress and function well in local environments (Canaza-Cayo *et al.*, 2015).

### Infectious disease and injury

Injury and disease are stresses that create strain that can affect production and pregnancy outcome. Routine vaccination programs control many of theinfectious diseases that negatively impact production and pregnancy (Newcomer and Givens, 2016). There are pathogens that affect postpartum reproduction that cannot be effectively controlled by vaccination (Gilbert, 2019). Infectious disease stress, therefore, is common in postpartum cows. Common diseases that affect postpartum dairy cows include metabolic diseases (ketosis and fatty liver) (Overton *et al.*, 2017), periparturient disorders (dystocia and retained placenta) (Funnell and Hilton, 2016), uterine diseases (metritis and endometritis) (Gilbert, 2019) and mastitis (Sordillo, 2018). Dystocia predisposes cattle to retained placenta and uterine infection (metritis) both of which are painful and are associated with reduced feed intake (Esposito *et al.*, 2014; Collier *et al.*, 2017; Shock *et al.*, 2018). Decreased feed intake can lead to metabolic and hormonal changes and associated weight loss (strain) that can affect pregnancy outcome. Likewise, cattle can become injured because of poor facility design on farm (for example, hock lesions and other abrasions in free stall barns) (Jewell *et al.*, 2019). Correct barn and stall design (including flooring) can reduce injury (a stress) and associated strain in lactating dairy cows (Cook, 2019; Kammel *et al.*, 2019).

### High milk production

Genetic selection within the dairy industry has successfully increased milk production per cow (Miglior *et al.*, 2017; Lucy, 2019). For the purpose of this review, high milk production will be defined as a stress. There is a change in circulating hormones and metabolites associated with high milk production and this creates a strain within the animal (Lucy *et al.*, 2014). The strain affects the normal function of the hypothatlamic-pituitary-gonadal (HPG) axis (abnormal gonadotropin secretion) that leads to ovarian dysfunction (Butler, 2000, 2014). Physiological stress caused by high milk production also affects the immune systems to create immune dysfunction (a strain) and disease (LeBlanc, 2012; Lucy *et al.*, 2014; Gilbert, 2019).

### Undernutrition

Under-feeding may arise from short or long-term feed shortages. Cows that are under-fed are stressed and undergo metabolic adaptation to the stress [lipid mobilization and non-esterified fatty acid (NEFA) release etc.] (D’Occhio *et al.*, 2019). There are similarities with respect to the hormonal and metabolic changes that occur in high milk-producing cows and underfed cows but there are differences as well. One important biological difference is that cows that are under-fed are mobilizing nutrients from tissue for survival. High producing cows that are fully-fed, however, are undergoing a genetically programmed homeorhetic process to support high milk production. Other nutritional stresses that include over-feeding protein (Butler, 1998) or under-feeding minerals (Wilde, 2006) can affect pregnancy outcome but will not be discussed further in this review.

### Psychological

Psychological stress may occur on farms (Dobson and Smith, 2000; von Keyserlingk and Weary, 2017). The common forms of psychological stress include social interactions with other farm animals and people (Lima *et al.*, 2018). Cattle are social animals that live in groups with a dominance hierarchy. Mixing groups of cattle creates stress. Productivity is decreased when the hypothalamic-pituitary- adrenal (HPA) axis is activated (Hong *et al.*, 2019) and feed intake is reduced (O’Driscoll *et al.*, 2006). Cows also spend time and energy to re-establish the dominance structure within the group (Val-Laillet *et al.*, 2008) and displace one another at the feed bunk (Huzzey *et al.*, 2006). Displacement will affect feeding behavior in subordinate cows. Cows that have recently calved and younger cows may benefit from being housed in smaller groups with less competition (Jensen and Proudfoot, 2017). In addition to other animals, cattle may experience stress from interaction with humans depending on how animals are handled and the specific individuals involved (Lima *et al.*, 2018).

## Mediators of stress in the whole animal

As stated in the introduction, a stress can be applied equally across animals. The strain is the response of the individual to the stress (Fig. 1). The amount of strain may be explained by the magnitude of the specific response (for example, how much NEFA is released postpartum) or the strain may be explained by the sensitivity of the animal to the specific response (for example, some cows have less strain because they are better able to metabolize NEFA). This section will describe some the mediators that link the stress to the strains that affect pregnancy outcome (Fig. 2).

### Elevated body temperature, systemic toxicity with fever, and chronic pain

Many of the effects of heat stress on pregnancy outcome can be explained by the increase in body temperature in heat-stressed cows. Small increases in maternal body temperature will decrease pregnancy rates in cattle (Ulberg and Burfening, 1967). The increase in body temperature affects the reproductive tract and the early embryo. One possible mechanism involves the direct effect of elevated temperature on the embryo (Hansen, 2009). A second mechanism involves the effect of heat stress on the gut (leaky gut syndrome) that causes loss of intestinal barrier function and the release of endotoxin into the circulation and may affect animal productivity and pregnancy outcome (Baumgard and Rhoads, 2013; Abuajamieh *et al.*, 2018).

Fever presents a change in hypothalamic body temperature set point in response to disease (Collier *et al.*, 2017). Disease with fever and injury with chronic pain will affect a cow’s motivation to eat or the capacity of the cow to reach the feed bunk (Aditya *et al.*, 2017). Poor intake causes a shift in hormones and metabolites toward a catabolic state (similar to that described in theprevious section). Disease releases endotoxins into the systemic circulation that can have direct effects on reproductive tissues themselves (Eckel and Ametaj, 2016) and also increase body temperature. For example, mastitis infection can cause endotoxin release, immune system activation, cytokine production, and body temperature elevation. The cumulative effects on the whole animal can damage the developing embryo and (or) cause regression of the corpus luteum and early embryonic loss (Hansen *et al.*, 2004; Kumar *et al.*, 2017).

### Metabolic and hormonal imbalance caused by inadequate nutrient intake

A high production cow in early lactation will produce over 50 kg of milk per day. The milk production represents a type of stress. In response to the stress, the cow undergoes homeorhesis; a term that was originally defined as “the orchestrated or coordinated control in metabolism of body tissues necessary to support a physiological state” (Bauman and Currie, 1980). There is an increase in circulating growth hormone (GH) postpartum that stimulates hepatic gluconeogenesis and increases glucose supply (Lucy, 2004; Baumgard *et al*., 2017). Growth hormone also antagonizes insulin action and creates an insulin resistant state (preventing the utilization of glucose by liver, muscle or adipose tissue). The increase in GH stimulates lipolysis that mobilizes fatty acids (NEFA) from adipose tissue. The large mass of glucose created through gluconeogenesis and fatty acids created though lipolysis are used for milk synthesis.

The strain associated with production stress is explained by changes in circulating hormone and metabolites. Some cows experience a larger hormonal and metabolic change that can lead to disease (for example, ketosis) (White, 2015). Low blood glucose concentrations postpartum are associated with low blood insulin concentrations. Low blood insulin is associated with low liver GH receptor expression and low circulating IGF1 concentrations (Lucy, 2004). Inadequate glucose supply contributes to the incomplete oxidation of fatty acids (NEFA) which creates elevated beta-hydroxybutyrate (BHB) postpartum (ketosis). The metabolic and endocrine state of early lactation (high GH, low IGF1, low insulin, low glucose, high NEFA and high BHB) affects the ability of the cow to become pregnant.

Cows that eat poorly because of disease or under-feeding undergo many of the same metabolic changes. There is an uncoupling of the somatotropic axis when animals are not eating (Radcliff *et al.*, 2003). Uncoupling of the axis leads to less IGF1 and elevated GH concentrations. The increase in GH drives lipid mobilization to increase NEFA in the circulation. Ketosis may occur if there is insufficient glucose supply and incomplete oxidation of fatty acids. There is also reduced circulating insulin associated with depressed circulating glucose and insulin resistance associated with elevated GH.

### HPG Axis function

Luteinizing hormone (LH) is a critical pituitary hormone for the resumption of normal estrous cycles in postpartum cows (Canfield and Butler, 1990, 1991). Greater frequency of LH pulses leads to maturation of preovulatory follicles and the initiation of cyclicity. Preovulatory follicles secrete estradiol that causes the hypothalamus to release a surge of GnRH to cause the LH surge for ovulation and formation of the corpus luteum. Stress can cause a strain on reproduction by slowing the pulsatile release of LH, decreasing follicular estradiol, and (or) blocking the LH surge (Karsch *et al.*, 2002; Breen and Karsch, 2006).

A variety of metabolites and metabolic signals can act at the level of the hypothalamus to affect GnRH and LH pulsatility. Glucose controls insulin secretion in the whole animal and ultimately controls hepatic IGF1 secretion via insulin release (Butler *et al.*, 2003). Circulating glucose and the insulin/IGF1 systems, therefore, are functionally linked in the whole animal (Lucy, 2004; Kawashima *et al.*, 2012). One study concluded that glucose and insulin were the most-likely molecules to exert a positive effect on hypothalamic GnRH and LH secretion in the postpartum dairy cow (Leroy *et al.*, 2008). The most important actions of insulin and IGF1 are observed when the hormone acts synergistically with the gonadotropins [stimulating hormone (FSH) or LH] (Lucy, 2011). There is a positive correlation between circulating concentrations of insulin and IGF1 and the interval to first postpartum ovulation (Velazquez *et al.*, 2008; Kawashima *et al.*, 2012). In a variety of species (including farm animals, humans, and laboratory animals) greater concentrations of blood IGF1 are found in young, well nourished, fertile, and healthy individuals (Thissen *et al.*, 1994). Animals that are old, diseased, infertile, or malnourished have low blood IGF1 concentrations.

### HPA/HPG Interactions

The hypothalamic-pituitary-adrenal (HPA) axis is activated in response to stress (Minton, 1994). The reproductive system can be affected by the HPA axis through interactions with the HPG axis (Collier *et al.*, 2017). Neurons within the hypothalamus secrete corticotropin releasing hormone (CRH) into the median eminence. The CRH travels through the hypothalamic-pituitary portal system and causes the release of ACTH from pituitary corticotroph cells. Adrenocorticotrophic hormone causes the adrenal gland to synthesize and secrete glucocorticoid (cortisol). The stressors that cause activation of the HPA axis may cause infertility by inhibiting LH secretion (Breen and Karsch, 2006). Some CRH neurons within the hypothalamus terminate on the cell bodies of GnRH neurons (Wade and Jones, 2004). When CRH neurons are stimulated and release CRH, GnRH release from GnRH neurons may be blocked. There are inhibitory effects of glucocorticoids on GnRH and LH release but these effects are not directly mediated by glucocorticoids because GnRH neurons do not possess the type II glucocorticoidreceptor. The KNDy cells within the hypothalamus that express kisspeptin, neurokinin B, and dynorphin do possess glucocorticoid receptor and may transmit the glucocorticoid signal to the GnRH neurons (Ralph *et al.,* 2016; Scott *et al.,* 2019) .

## Mechanisms through which stress and strain affect pregnancy outcome

Stress and strain affect pregnancy outcome. In some cases, the stress can act directly on the pregnancy itself. For example when elevated body temperature from heat stress affects ovarian function, developmental capacity of the oocyte, or early pregnancy development. There are other examples where a strain affects pregnancy outcome. An example would be when hormonal and metabolic imbalance postpartum causes immune dysfunction that leads to uterine disease and infertility. Mechanisms through which stress and strain affect pregnancy outcome are discussed in this section.

### Elevated body temperature (heat stress)

Many investigators have reported reduced estrogenic capacity of the ovarian follicle in response to heat stress (Gwazdauskas, 1985; Wolfenson *et al.*, 1997; Wilson *et al.*, 1998). The somatic cells within the follicle (theca and granulosa cells), therefore, can be damaged when cows have elevated body temperature caused by heat stress. Whether or not heat stress affects the corpus luteum is less clear (Hansen and Aréchiga, 1999). The cells of the corpus luteum differentiate from the cells of the follicle. If heat stress decreases blood progesterone then the decrease could arise from the effects of heat stress on the follicle which ultimately forms the corpus luteum.

There are large and consistent effects of heat stress on the oocyte and developing embryo (Putney *et al.*, 1989; Ealy *et al.*, 1993; Hansen, 2009; Roth, 2018a, b). The period of greatest susceptibility of the oocyte/embryo is immediately after the onset of estrus and early during the post-breeding period (Sakatani, 2017). Embryonic development was impaired in heifers subjected to heat stress for 10 hours after the onset of estrus (Putney *et al.*, 1989). Heat stress on day 1 after breeding also decreased subsequent embryonic development. Heat stress on days 3, 5, or 7 after breeding, however, did not affect embryonic development (Ealy *et al.*, 1993). The period of embryonic sensitivity to heat stress, therefore, begins early during the development of the follicle and continues until about 1 day after breeding. By 3 days after breeding, embryos have apparently developed resistance to the effects of heat stress. Several investigators have demonstrated that embryo transfer nearly doubled conception rates when compared with dairy cows inseminated artificially at estrus (Hansen, 2007). It is possible, therefore, to by-pass early embryonic stages and improve conception rates during heat stress.

### Metabolic imbalance postpartum

The associations between postpartum hormones and metabolites and subsequent reproduction are found early postpartum when the most-extreme homeorhetic states are known to occur. The early postpartum metabolic profile, therefore, may have the capacity to imprint ovarian tissue either through permanent effects on the genome (epigenetic mechanisms) or by changing the chemical composition of the cells themselves. The oocyte rests in a quiescent state within the ovary until approximately 2 months before ovulation. At that time, it initiates growth along with the surrounding granulosa cells. The metabolic environment within which the oocyte develops can affect its capacity for fertilization and further development (Leroy *et al.*, 2008, 2011; Berlinguer *et al.*, 2012). One theory is that the long development program of the oocyte before ovulation enables an irreversible imprinting of the metabolome on the oocyte itself. If this imprint is negative then this may explain why cows with metabolic disease early postpartum have infertility several months later.

Glucose is an important energy source for ATP production through mitochondrial oxidative phosphorylation. In the uterus and placenta, however, the bulk of the glucose is used to supply carbons for the synthesis of cellular components (nucleotides, amino acids, lipids, etc.). This latter phenomenon is known as the “Warburg effect” and typifies proliferating cells (Vander Heiden *et al.*, 2009). In a study designed to test the effects of glucose on the pregnancy, cows were either milked normally or dried off (not milked) immediately after calving (Green *et al.*, 2012). Milking or not milking postpartum created treatment groups with either low or high circulating glucose concentrations, respectively. The fetus and placenta from the milked (lactating) cows were smaller (weighed less) than the fetus and placenta from nonlactating cows. There was less glucose reaching the fetus in lactating compared with nonlactating cows (Lucy *et al.*, 2012). The reduction in glucose reaching the pregnancy could potentially affect how the pregnancy develops because the pregnancy depends on glucose as a substrate for tissue synthesis and metabolic energy (Battaglia and Meschia, 1978). In the horse, delayed development of the embryonic vesicle generally leads to embryonic loss (Carnevale *et al.*, 2000). Several recent studies in the bovine have demonstrated that pregnant cows that undergo pregnancy loss have lesser blood concentrations of pregnancy associated glycoproteins (PAG) leading up to the time that the pregnancy is aborted (Pohler *et al.*, 2016). The lesser blood PAG concentration may indicate that the cow is pregnant with a small embryo or fetus. Low concentrations of glucose in postpartum cows, therefore, may predispose the cow to pregnancy loss if the placenta does not have adequate substrate for the creation of new cells and the pregnancy grows too slowly (Lucy *et al.*, 2014).

### Immune dysfunction

The strain of an abnormal metabolic and hormonal environment postpartum creates dysfunction within the innate immune system through its effects on polymorphonuclear neutrophils (PMN) (Lucy, 2004; Graugnard *et al.*, 2012; LeBlanc, 2012). For example, glucose is the primary metabolic fuel that PMN use to generate the oxidative burst that leads to killing activity. Glycogen concentrations in PMN within the postpartum cow decrease in a manner that is similar to the decrease in blood glucose postpartum (Galvão *et al.*, 2010). Galvão *et al.* (2010) concluded that the lesser glycogen reserve reduced the capacity for oxidative burst in PMN and predisposed the cow to uterine disease.

Epidemiological evidence indicates that an abnormal metabolic profile during the periparturient period predisposes the cow to uterine disease during the early postpartum period and infertility later postpartum (Chapinal *et al.*, 2012; Wathes, 2012; Gilbert, 2019). Cows that had uterine infection early postpartum have more inflammation in the pregnant uterus (Lucy *et al.*, 2016). Inflammation and the presence of lymphocytic foci within the pregnant uterus were associated with a smaller placenta and embryonic loss (Lucy *et al.*, 2016).

### Ovarian dysfunction

Stressors that affect ovarian function in dairy cattle commonly do so by interfering with LH release. For example, negative energy balance in dairy cattle (a stress) will cause a decrease in the frequency of LH pulses (a strain) (Canfield and Butler, 1990, 1991). The exact mechanisms through which undernutrition slows the frequency of LH pulses are poorly understood but a variety of mechanisms are probably involved.

Follicular growth and steroidogenesis in postpartum cattle depends on the combined effects of gonadotropins (LH and FSH), systemic hormones (insulin and IGF1) and metabolites (glucose) whose concentrations are highly correlated (Lucy, 2004). The magnitude and duration of the decrease in circulating insulin and IGF1 depends on the depth of negative energy balance postpartum (Beam and Butler, 1999). Cattle in poor body condition or cows failing to increase body condition during lactation have an extended period of low blood insulin and IGF1 and elevated blood GH. There is a positive correlation between blood insulin and IGF1 concentrations and ovarian function in postpartum cows (Wathes *et al.*, 2007). Greater LH pulsatility leads to increased follicular growth that decreases the interval to first ovulation. Both insulin and IGF1 may control the activity of the GnRH neurons in the hypothalamus and (or) the LH release from gonadotrophs (Veldhuis *et al.*, 2006).

Patterns of estrous cyclicity for lactating cows are less regular when compared with estrous cycle of nulliparous heifers (Lucy, 2019). The same hormones that control when the cow begins to cycle (insulin, IGF1, and LH) also have an effect on cyclicity which relates to the functionality of the follicle and corpus luteum. The hormonal environment created by lactation (in this example low blood glucose, insulin and IGF1 concentrations) may potentially affect the capacity for ovarian cells to respond to gonadotropins. In the cycling cow, this could potentially affect estradiol production by the follicle as well as progesterone production by the corpus luteum. Low blood glucose could potentially compromise a variety of essential metabolic processes in ovarian cells including the oocyte that depends on glucose for energy (Berlinguer *et al.*, 2012). There is also the potential for greater steroid metabolism in lactating compared with nonlactating cows that can be explained by greater dry matter intake in cows that are lactating (Wiltbank *et al.*, 2011). Lesser circulating estradiol from the preovulatory follicle can lead to abnormal patterns of follicular growth, anovulatory conditions, multiple ovulation and also reduced estrous expression (Lucy, 2019).

Several authors have recently reviewed the mechanisms assoicated with subnormal luteal development and early embryonic loss (Spencer *et al.*, 2016; Forde and Lonergan, 2017). Low progesterone during the first weeks after insemination may be caused by the stress of high milk production (Lonergan, 2011; Wiltbank *et al.*, 2011). Progesterone stimulates uterine histotroph secretion and lesser uterine histotroph secretion (caused by low progresteorne) may lead to slower embyronic development. The slowly developing embryos may fail to reach adequate size to generate an adequate interferon-tau (IFNT) signal to the mother (Hansen *et al.*, 2017). The pregnancy is lost because the mother fails to recognize the pregnancy and undergoes luteal regression as if she is not pregnant.

Immune dysfunction postpartum may be associated with a high incidence of mastitis in early postpartum cows (Sordillo, 2018). Mastitis may not directly affect reproductive tissues but secondary responses of the cow to the disease can disrupt estrous cycles and cause embryonic loss. Several authors have found that a mastitis event during breeding was associated with lower fertility (Fuenzalida *et al.*, 2015). Cytokines and other hormones released by the inflamed mammary tissue can circulate throughout the cow and block ovulation or cause premature regression of the corpus luteum (Sheldon, 2015).

### Psychological stress

Cows interact with other cows in the herd and also the people that care for them. The mechanisms linking changes in social status within the herd to reproductive efficiency are not clear but may involve activation of the HPA axis and subsequent inhibition of the HPG axis in animals that are subjected to aggression from other cows. A recent study of dairy cows showed that dairy cows losing social status during the breeding period had a longer interval from calving to conception and required more inseminations per conception (Dobson and Smith, 2000). The activation of the HPA axis that occurs in response to social stress can inhibit the pituitary release of LH (Karsch *et al.*, 2002). Furthermore, cortisol may decrease responsiveness of ovarian follicles to LH.

Dairy cattle can recognize individual people and have better performance when handled by gentle people compared with aggressive people (Munksgaard *et al.*, 1997; Lindahl *et al.*, 2016). Conception rate was positively correlated with positive human-animal interactions in one study of 66 commercial farms (Hemsworth *et al.*, 2000). It is possible that some of the variation in inseminator conception rate could be explained by handling of the animals before and during insemination. Aggressive handling may activate the HPA axis and disrupt normal processes that precede ovulation and affect fertility.

## Conclusions

Stress and associated strain are important topics because they affect the ability of farm animals to become pregnant. Stresses arise from a variety of sources that reside outside or within the individual animal. Outside sources of stress include the physical environment (ambient temperature and humidity), physical surroundings (facilities), other cows (social interactions), people (human-cattle interactions) and microbial (disease). To some extent, the strain from outside sources can be mitigated by reducing the stress itself. For example, the physical environment can be improved by cooling, facilities can be improved by replacement or renovation, cows can be housed in smaller groups of similar-sized cattle, aggressive cow handlers can be re-trained so that they use appropriate techniques, and disease can be reduced through vaccination, cleanliness, and antibiotic treatment. Stress can also come from within the animal (abnormal thermal, hormonal or metabolic profile that creates immunological and ovarian dysfunction, uterine disease, poor oocyte quality and embryonic loss). The strain from the internal stress response can be managed through programs such as timed AI that control ovarian function and the time of breeding (Carvalho *et al*., 2018) or embryo transfer that circumvents periods of embryo sensitivity (Hansen, 2007). Genetic selection of animals that are resistant to stress and have less strain is an additional method to improve productivity. The goal for future of management and genetic selection programs in farm animals should be to reduce production stress, manage the remaining strain using technologies like timed AI and embryo transfer and also genetically select cattle with minimal strain in response to stress.
